# 1-Chloro­acetyl-2,6-bis­(2-chloro­phen­yl)-3,5-dimethyl­piperidin-4-one

**DOI:** 10.1107/S1600536810031247

**Published:** 2010-08-11

**Authors:** R. Ramachandran, P. Parthiban, M. Rani, S. Kabilan, Yeon Tae Jeong

**Affiliations:** a Department of Image Science and Engineering, Pukyong National University, Busan 608-739, Republic of Korea; b Department of Chemistry, Annamalai University, Annamalai Nagar 608 002, Tamil Nadu, India

## Abstract

In the title compound, C_21_H_20_Cl_3_NO_2_, the piperidin-4-one ring adopts a boat conformation. The two 2-chloro­phenyl groups are approximately perpendicular to each other, making a dihedral angle of 74.07 (8)°.

## Related literature

For the biological activity of related structures, see: Parthiban *et al.* (2009 ▶); Aridoss *et al.* (2007 ▶). For spectroscopic studies of piperidin-4-ones, see: Ravindran *et al.* (1991 ▶); Krishnakumar *et al.* (1996 ▶). For ring conformational analysis, see: Cremer & Pople (1975 ▶); Nardelli (1983 ▶). For the synthesis of the title compound, see: Ramachandran *et al.* (2008 ▶); Aridoss *et al.* (2010 ▶).
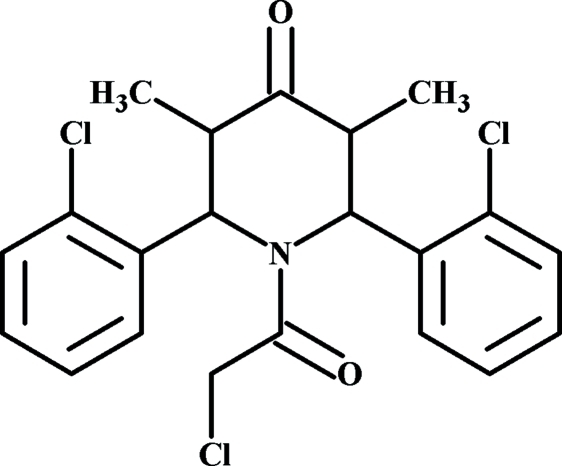


## Experimental

### 

#### Crystal data

C_21_H_20_Cl_3_NO_2_
*M* *_r_* = 424.73
Monoclinic, 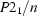

*a* = 11.6295 (4) Å
*b* = 9.6955 (3) Å
*c* = 17.4743 (5) Å
β = 90.481 (1)°
*V* = 1970.22 (11) Å^3^
*Z* = 4
Mo *K*α radiation
μ = 0.48 mm^−1^
*T* = 293 K
0.22 × 0.16 × 0.16 mm

#### Data collection

Bruker Kappa APEXII CCD diffractometer
Absorption correction: multi-scan (Blessing, 1995 ▶) *T*_min_ = 0.901, *T*_max_ = 0.927
27536 measured reflections
6864 independent reflections
4998 reflections with *I* > 2σ(*I*)
*R*_int_ = 0.023

#### Refinement

*R*[*F* ^2^ > 2σ(*F* ^2^)] = 0.045
*wR*(*F* ^2^) = 0.141
*S* = 1.01
6864 reflections
244 parameters
H-atom parameters constrained
Δρ_max_ = 0.40 e Å^−3^
Δρ_min_ = −0.31 e Å^−3^

### 

Data collection: *APEX2* (Bruker, 2004 ▶); cell refinement: *APEX2* and *SAINT* (Bruker, 2004 ▶); data reduction: *SAINT* and *XPREP* (Bruker, 2004 ▶); program(s) used to solve structure: *SIR92* (Altomare *et al.*, 1993 ▶); program(s) used to refine structure: *SHELXL97* (Sheldrick, 2008 ▶); molecular graphics: *ORTEP-3* (Farrugia, 1997 ▶); software used to prepare material for publication: *SHELXL97*.

## Supplementary Material

Crystal structure: contains datablocks global, I. DOI: 10.1107/S1600536810031247/ez2226sup1.cif

Structure factors: contains datablocks I. DOI: 10.1107/S1600536810031247/ez2226Isup2.hkl

Additional supplementary materials:  crystallographic information; 3D view; checkCIF report

## Figures and Tables

**Fig. 1.**
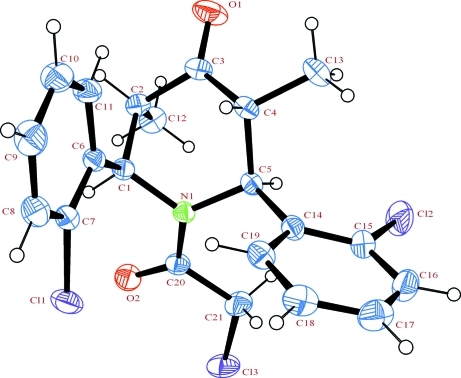
The molecular structure of title compound, showing 50% probability displacement ellipsoids.

## supplementary crystallographic information

### Comment

2,6-Disubstituted piperidones and their N-substituted compounds are of great interest due to their significant pharmacological properties (Parthiban *et al.*, 2009; Aridoss *et al.*, 2007). The introduction of electron withdrawing groups such as –CHO, COCH_3_, COPh, NO, *etc*., at the ring nitrogen cause a major change in ring conformation (Ravindran *et al.*, 1991; Krishnakumar *et al.*, 1996). Hence, we introduced the chloroacetyl (COCH_2_Cl) group into the piperidine ring in order to analyse the ring conformation through a single-crystal X-ray diffraction study.

In the molecular structure (C_21_H_20_Cl_2_NO_2_), the piperidine ring adopts a boat conformation with puckering parameters (Cremer & Pople, 1975) as follows: Total puckering amplitude, Q_T_=0.6960 (15)Å and phase angle θ=85.52 (12)°. The smallest displacement asymmetry parameters (Nardelli, 1983) q_1_ and q_2_ are 0.6939 (15) Å and 0.0544 (15) Å, respectively. The dihedral angle between the two *o*-chlorophenyl rings is 74.07 (8) °.

### Experimental

The title compound was obtained by adopting an earlier method (Ramachandran *et al.* (2008); Aridoss *et al.*, 2010). To a well stirred solution of 3,5-dimethyl-2,6-bis(*o*-chloroyphenyl)piperidin-4-one (2 g, 4.71 mmol) and triethylamine (1.42 g, 14.13 mmol) in freshly distilled benzene (50 ml), chloroacetyl chloride (0.79 g, 7.06 mmol) in benzene (10 ml) was added drop-wise through the addition funnel over about half an hour. Stirring was continued until the completion of reaction. The reaction mixture was then poured into water and extracted with DCM. The solvent was removed under reduced pressure. The crude sample was purified by column chromatography. Upon recrystallization from absolute ethanol this afforded fine white crystals suitable for X-ray diffraction analysis.

### Refinement

H-atoms were positioned and refined using a riding model, with aromatic C—H = 0.93 Å, methine C—H = 0.98 Å, methylene C—H = 0.97 Å and methyl C—H = 0.96 Å. The displacement parameters were set for phenyl, methylene and aliphatic H atoms at *U*_iso_(H)=1.2*U*_eq_(C) or 1.5_eq_(methyl C).

### Crystal data

**Table:**

C_21_H_20_Cl_3_NO_2_	*F*(000) = 880
*M**_r_* = 424.73	*D*_x_ = 1.432 Mg m^−^^3^
Monoclinic, *P*2_1_/*n*	Mo *K*α radiation, λ = 0.71073 Å
Hall symbol: -P 2yn	Cell parameters from 5467 reflections
*a* = 11.6295 (4) Å	θ = 2.1–25.0°
*b* = 9.6955 (3) Å	µ = 0.48 mm^−^^1^
*c* = 17.4743 (5) Å	*T* = 293 K
β = 90.481 (1)°	Prism, colourless
*V* = 1970.22 (11) Å^3^	0.22 × 0.16 × 0.16 mm
*Z* = 4	

### Data collection

**Table:**

Bruker Kappa APEXII CCD diffractometer	6864 independent reflections
Radiation source: fine-focus sealed tube	4998 reflections with *I* > 2σ(*I*)
graphite	*R*_int_ = 0.023
ω and φ scan	θ_max_ = 32.1°, θ_min_ = 2.1°
Absorption correction: multi-scan (Blessing, 1995)	*h* = −17→17
*T*_min_ = 0.901, *T*_max_ = 0.927	*k* = −14→14
27536 measured reflections	*l* = −26→26

### Refinement

**Table:**

Refinement on *F*^2^	Primary atom site location: structure-invariant direct methods
Least-squares matrix: full	Secondary atom site location: difference Fourier map
*R*[*F*^2^ > 2σ(*F*^2^)] = 0.045	Hydrogen site location: inferred from neighbouring sites
*wR*(*F*^2^) = 0.141	H-atom parameters constrained
*S* = 1.01	*w* = 1/[σ^2^(*F*_o_^2^) + (0.0727*P*)^2^ + 0.5226*P*] where *P* = (*F*_o_^2^ + 2*F*_c_^2^)/3
6864 reflections	(Δ/σ)_max_ = 0.001
244 parameters	Δρ_max_ = 0.40 e Å^−^^3^
0 restraints	Δρ_min_ = −0.31 e Å^−^^3^

### Special details

**Table:**

Geometry. All e.s.d.'s (except the e.s.d. in the dihedral angle between two l.s. planes) are estimated using the full covariance matrix. The cell e.s.d.'s are taken into account individually in the estimation of e.s.d.'s in distances, angles and torsion angles; correlations between e.s.d.'s in cell parameters are only used when they are defined by crystal symmetry. An approximate (isotropic) treatment of cell e.s.d.'s is used for estimating e.s.d.'s involving l.s. planes.
Refinement. Refinement of *F*^2^ against ALL reflections. The weighted *R*-factor *wR* and goodness of fit *S* are based on *F*^2^, conventional *R*-factors *R* are based on *F*, with *F* set to zero for negative *F*^2^. The threshold expression of *F*^2^ > σ(*F*^2^) is used only for calculating *R*-factors(gt) *etc*. and is not relevant to the choice of reflections for refinement. *R*-factors based on *F*^2^ are statistically about twice as large as those based on *F*, and *R*- factors based on ALL data will be even larger.

### Fractional atomic coordinates and isotropic or equivalent isotropic displacement parameters (Å^2^)

**Table:**

	*x*	*y*	*z*	*U*_iso_*/*U*_eq_	
C1	0.85514 (12)	0.37161 (13)	−0.00740 (8)	0.0341 (3)	
H1A	0.8746	0.4692	−0.0004	0.041*	
C2	0.96025 (12)	0.30363 (15)	−0.04343 (8)	0.0385 (3)	
H2A	1.0237	0.3032	−0.0062	0.046*	
C3	0.93368 (13)	0.15720 (15)	−0.06701 (9)	0.0415 (3)	
C4	0.80789 (13)	0.11575 (13)	−0.07058 (8)	0.0368 (3)	
H4A	0.7839	0.0981	−0.0178	0.044*	
C5	0.73061 (12)	0.23387 (13)	−0.10157 (7)	0.0340 (2)	
H5A	0.7456	0.2437	−0.1564	0.041*	
C6	0.82292 (12)	0.31664 (14)	0.07135 (7)	0.0346 (3)	
C7	0.73896 (13)	0.37879 (15)	0.11624 (8)	0.0393 (3)	
C8	0.70953 (16)	0.32934 (19)	0.18766 (9)	0.0505 (4)	
H8A	0.6519	0.3724	0.2155	0.061*	
C9	0.76589 (18)	0.2157 (2)	0.21762 (9)	0.0562 (4)	
H9A	0.7457	0.1810	0.2653	0.067*	
C10	0.85181 (17)	0.15487 (18)	0.17638 (10)	0.0529 (4)	
H10A	0.8911	0.0794	0.1965	0.063*	
C11	0.88047 (14)	0.20513 (16)	0.10491 (9)	0.0432 (3)	
H11A	0.9400	0.1633	0.0783	0.052*	
C12	0.99879 (15)	0.37953 (19)	−0.11617 (10)	0.0494 (4)	
H12A	1.0647	0.3339	−0.1371	0.074*	
H12B	1.0184	0.4731	−0.1036	0.074*	
H12C	0.9373	0.3790	−0.1532	0.074*	
C13	0.79149 (17)	−0.01856 (16)	−0.11449 (11)	0.0516 (4)	
H13A	0.8409	−0.0882	−0.0931	0.077*	
H13B	0.8104	−0.0044	−0.1673	0.077*	
H13C	0.7129	−0.0478	−0.1108	0.077*	
C14	0.60451 (13)	0.19672 (14)	−0.09241 (8)	0.0380 (3)	
C15	0.53718 (15)	0.14574 (17)	−0.15177 (10)	0.0491 (4)	
C16	0.42350 (17)	0.1070 (2)	−0.14119 (13)	0.0619 (5)	
H16A	0.3809	0.0702	−0.1816	0.074*	
C17	0.37424 (16)	0.12352 (19)	−0.07036 (14)	0.0614 (5)	
H17A	0.2978	0.0989	−0.0630	0.074*	
C18	0.43777 (16)	0.17623 (19)	−0.01064 (12)	0.0548 (4)	
H18A	0.4041	0.1889	0.0369	0.066*	
C19	0.55201 (14)	0.21042 (16)	−0.02132 (9)	0.0432 (3)	
H19A	0.5949	0.2434	0.0199	0.052*	
C20	0.73202 (12)	0.49115 (14)	−0.09783 (8)	0.0379 (3)	
C21	0.63849 (16)	0.48474 (18)	−0.15890 (10)	0.0514 (4)	
H21A	0.5701	0.4434	−0.1374	0.062*	
H21B	0.6640	0.4265	−0.2006	0.062*	
O1	1.01004 (12)	0.07846 (14)	−0.08337 (10)	0.0692 (4)	
O2	0.77569 (11)	0.59995 (11)	−0.07971 (7)	0.0528 (3)	
N1	0.75946 (10)	0.36719 (11)	−0.06397 (6)	0.0326 (2)	
Cl1	0.66924 (4)	0.52622 (4)	0.08431 (3)	0.05574 (13)	
Cl2	0.59251 (5)	0.13268 (7)	−0.24418 (3)	0.07505 (18)	
Cl3	0.60408 (4)	0.64954 (5)	−0.19502 (3)	0.06205 (14)	

### Atomic displacement parameters (Å^2^)

**Table:**

	*U*^11^	*U*^22^	*U*^33^	*U*^12^	*U*^13^	*U*^23^
C1	0.0384 (6)	0.0286 (6)	0.0353 (6)	0.0014 (5)	−0.0026 (5)	0.0020 (4)
C2	0.0353 (6)	0.0382 (7)	0.0421 (7)	0.0015 (5)	0.0007 (5)	0.0047 (5)
C3	0.0434 (7)	0.0362 (7)	0.0452 (7)	0.0071 (6)	0.0061 (6)	0.0042 (5)
C4	0.0440 (7)	0.0285 (6)	0.0379 (6)	0.0028 (5)	0.0056 (5)	−0.0003 (5)
C5	0.0386 (6)	0.0313 (6)	0.0320 (6)	0.0013 (5)	0.0025 (5)	−0.0023 (4)
C6	0.0407 (6)	0.0305 (6)	0.0325 (6)	0.0025 (5)	−0.0022 (5)	−0.0009 (5)
C7	0.0441 (7)	0.0364 (6)	0.0373 (6)	0.0048 (5)	−0.0030 (5)	−0.0051 (5)
C8	0.0543 (9)	0.0583 (9)	0.0390 (7)	0.0055 (7)	0.0056 (7)	−0.0077 (7)
C9	0.0723 (11)	0.0619 (10)	0.0345 (7)	0.0008 (9)	0.0044 (7)	0.0058 (7)
C10	0.0677 (11)	0.0490 (9)	0.0419 (8)	0.0094 (8)	−0.0027 (7)	0.0106 (6)
C11	0.0509 (8)	0.0402 (7)	0.0386 (7)	0.0104 (6)	−0.0005 (6)	0.0041 (6)
C12	0.0462 (8)	0.0539 (9)	0.0482 (8)	−0.0054 (7)	0.0069 (7)	0.0089 (7)
C13	0.0646 (10)	0.0333 (7)	0.0571 (9)	0.0017 (7)	0.0077 (8)	−0.0078 (6)
C14	0.0404 (7)	0.0328 (6)	0.0410 (7)	−0.0012 (5)	0.0011 (5)	−0.0040 (5)
C15	0.0493 (8)	0.0474 (8)	0.0504 (8)	−0.0004 (7)	−0.0060 (7)	−0.0089 (7)
C16	0.0525 (10)	0.0523 (10)	0.0804 (13)	−0.0082 (8)	−0.0200 (9)	−0.0034 (9)
C17	0.0431 (9)	0.0483 (9)	0.0929 (15)	−0.0075 (7)	0.0031 (9)	0.0139 (9)
C18	0.0503 (9)	0.0458 (8)	0.0686 (11)	−0.0028 (7)	0.0156 (8)	0.0093 (8)
C19	0.0464 (8)	0.0377 (7)	0.0455 (7)	−0.0033 (6)	0.0074 (6)	−0.0003 (6)
C20	0.0412 (7)	0.0346 (6)	0.0379 (6)	0.0049 (5)	0.0015 (5)	0.0057 (5)
C21	0.0559 (9)	0.0466 (8)	0.0516 (9)	0.0061 (7)	−0.0126 (7)	0.0126 (7)
O1	0.0535 (7)	0.0504 (7)	0.1039 (12)	0.0162 (6)	0.0167 (7)	−0.0077 (7)
O2	0.0664 (8)	0.0322 (5)	0.0596 (7)	−0.0005 (5)	−0.0111 (6)	0.0084 (5)
N1	0.0375 (5)	0.0277 (5)	0.0325 (5)	0.0015 (4)	−0.0017 (4)	0.0018 (4)
Cl1	0.0653 (3)	0.0465 (2)	0.0555 (2)	0.02331 (18)	0.00334 (19)	−0.00359 (17)
Cl2	0.0761 (3)	0.1018 (4)	0.0471 (2)	0.0034 (3)	−0.0109 (2)	−0.0279 (2)
Cl3	0.0654 (3)	0.0601 (3)	0.0607 (3)	0.0222 (2)	−0.0028 (2)	0.0202 (2)

### Geometric parameters (Å, °)

**Table:**

C1—N1	1.4828 (17)	C11—H11A	0.9300
C1—C6	1.5253 (18)	C12—H12A	0.9600
C1—C2	1.5291 (19)	C12—H12B	0.9600
C1—H1A	0.9800	C12—H12C	0.9600
C2—C3	1.510 (2)	C13—H13A	0.9600
C2—C12	1.539 (2)	C13—H13B	0.9600
C2—H2A	0.9800	C13—H13C	0.9600
C3—O1	1.2072 (18)	C14—C15	1.386 (2)
C3—C4	1.518 (2)	C14—C19	1.395 (2)
C4—C13	1.523 (2)	C15—C16	1.388 (3)
C4—C5	1.5506 (19)	C15—Cl2	1.7480 (19)
C4—H4A	0.9800	C16—C17	1.378 (3)
C5—N1	1.4871 (17)	C16—H16A	0.9300
C5—C14	1.520 (2)	C17—C18	1.372 (3)
C5—H5A	0.9800	C17—H17A	0.9300
C6—C7	1.3946 (19)	C18—C19	1.383 (2)
C6—C11	1.3979 (19)	C18—H18A	0.9300
C7—C8	1.383 (2)	C19—H19A	0.9300
C7—Cl1	1.7333 (15)	C20—O2	1.2117 (19)
C8—C9	1.382 (3)	C20—N1	1.3760 (16)
C8—H8A	0.9300	C20—C21	1.519 (2)
C9—C10	1.370 (3)	C21—Cl3	1.7628 (16)
C9—H9A	0.9300	C21—H21A	0.9700
C10—C11	1.384 (2)	C21—H21B	0.9700
C10—H10A	0.9300		
			
N1—C1—C6	113.75 (11)	C6—C11—H11A	118.9
N1—C1—C2	108.12 (11)	C2—C12—H12A	109.5
C6—C1—C2	115.10 (11)	C2—C12—H12B	109.5
N1—C1—H1A	106.4	H12A—C12—H12B	109.5
C6—C1—H1A	106.4	C2—C12—H12C	109.5
C2—C1—H1A	106.4	H12A—C12—H12C	109.5
C3—C2—C1	110.81 (12)	H12B—C12—H12C	109.5
C3—C2—C12	106.54 (13)	C4—C13—H13A	109.5
C1—C2—C12	111.93 (12)	C4—C13—H13B	109.5
C3—C2—H2A	109.2	H13A—C13—H13B	109.5
C1—C2—H2A	109.2	C4—C13—H13C	109.5
C12—C2—H2A	109.2	H13A—C13—H13C	109.5
O1—C3—C2	120.67 (15)	H13B—C13—H13C	109.5
O1—C3—C4	122.25 (15)	C15—C14—C19	116.86 (14)
C2—C3—C4	117.08 (12)	C15—C14—C5	123.05 (13)
C3—C4—C13	111.32 (13)	C19—C14—C5	120.08 (13)
C3—C4—C5	111.98 (11)	C14—C15—C16	121.98 (17)
C13—C4—C5	112.70 (13)	C14—C15—Cl2	120.50 (13)
C3—C4—H4A	106.8	C16—C15—Cl2	117.50 (14)
C13—C4—H4A	106.8	C17—C16—C15	119.46 (18)
C5—C4—H4A	106.8	C17—C16—H16A	120.3
N1—C5—C14	111.94 (10)	C15—C16—H16A	120.3
N1—C5—C4	111.08 (11)	C18—C17—C16	120.09 (17)
C14—C5—C4	110.19 (11)	C18—C17—H17A	120.0
N1—C5—H5A	107.8	C16—C17—H17A	120.0
C14—C5—H5A	107.8	C17—C18—C19	119.88 (18)
C4—C5—H5A	107.8	C17—C18—H18A	120.1
C7—C6—C11	115.67 (13)	C19—C18—H18A	120.1
C7—C6—C1	122.34 (12)	C18—C19—C14	121.68 (16)
C11—C6—C1	121.90 (12)	C18—C19—H19A	119.2
C8—C7—C6	122.53 (14)	C14—C19—H19A	119.2
C8—C7—Cl1	117.25 (12)	O2—C20—N1	123.57 (13)
C6—C7—Cl1	120.20 (11)	O2—C20—C21	121.02 (13)
C9—C8—C7	119.87 (15)	N1—C20—C21	115.37 (13)
C9—C8—H8A	120.1	C20—C21—Cl3	111.94 (12)
C7—C8—H8A	120.1	C20—C21—H21A	109.2
C10—C9—C8	119.31 (15)	Cl3—C21—H21A	109.2
C10—C9—H9A	120.3	C20—C21—H21B	109.2
C8—C9—H9A	120.3	Cl3—C21—H21B	109.2
C9—C10—C11	120.33 (16)	H21A—C21—H21B	107.9
C9—C10—H10A	119.8	C20—N1—C1	115.56 (11)
C11—C10—H10A	119.8	C20—N1—C5	121.24 (11)
C10—C11—C6	122.20 (15)	C1—N1—C5	119.01 (10)
C10—C11—H11A	118.9		
			
N1—C1—C2—C3	−58.03 (14)	C1—C6—C11—C10	179.89 (15)
C6—C1—C2—C3	70.37 (15)	N1—C5—C14—C15	136.19 (14)
N1—C1—C2—C12	60.74 (15)	C4—C5—C14—C15	−99.67 (16)
C6—C1—C2—C12	−170.85 (12)	N1—C5—C14—C19	−45.29 (17)
C1—C2—C3—O1	−166.27 (15)	C4—C5—C14—C19	78.86 (16)
C12—C2—C3—O1	71.75 (19)	C19—C14—C15—C16	−1.4 (2)
C1—C2—C3—C4	14.92 (17)	C5—C14—C15—C16	177.20 (16)
C12—C2—C3—C4	−107.06 (14)	C19—C14—C15—Cl2	176.79 (12)
O1—C3—C4—C13	−13.9 (2)	C5—C14—C15—Cl2	−4.6 (2)
C2—C3—C4—C13	164.91 (13)	C14—C15—C16—C17	2.2 (3)
O1—C3—C4—C5	−141.05 (16)	Cl2—C15—C16—C17	−176.06 (15)
C2—C3—C4—C5	37.74 (17)	C15—C16—C17—C18	−0.8 (3)
C3—C4—C5—N1	−46.40 (15)	C16—C17—C18—C19	−1.2 (3)
C13—C4—C5—N1	−172.84 (12)	C17—C18—C19—C14	2.0 (3)
C3—C4—C5—C14	−171.04 (11)	C15—C14—C19—C18	−0.7 (2)
C13—C4—C5—C14	62.52 (15)	C5—C14—C19—C18	−179.31 (14)
N1—C1—C6—C7	−62.84 (17)	O2—C20—C21—Cl3	−1.2 (2)
C2—C1—C6—C7	171.62 (13)	N1—C20—C21—Cl3	176.76 (11)
N1—C1—C6—C11	120.85 (14)	O2—C20—N1—C1	−5.4 (2)
C2—C1—C6—C11	−4.69 (19)	C21—C20—N1—C1	176.75 (13)
C11—C6—C7—C8	−3.4 (2)	O2—C20—N1—C5	−162.10 (14)
C1—C6—C7—C8	−179.97 (14)	C21—C20—N1—C5	20.01 (19)
C11—C6—C7—Cl1	174.97 (12)	C6—C1—N1—C20	123.99 (12)
C1—C6—C7—Cl1	−1.55 (19)	C2—C1—N1—C20	−106.85 (13)
C6—C7—C8—C9	1.4 (3)	C6—C1—N1—C5	−78.72 (14)
Cl1—C7—C8—C9	−177.11 (14)	C2—C1—N1—C5	50.45 (14)
C7—C8—C9—C10	1.0 (3)	C14—C5—N1—C20	−78.22 (15)
C8—C9—C10—C11	−1.1 (3)	C4—C5—N1—C20	158.14 (12)
C9—C10—C11—C6	−1.2 (3)	C14—C5—N1—C1	125.81 (12)
C7—C6—C11—C10	3.4 (2)	C4—C5—N1—C1	2.17 (16)
